# A Deep-Learning Approach to Spleen Volume Estimation in Patients with Gaucher Disease

**DOI:** 10.3390/jcm12165361

**Published:** 2023-08-18

**Authors:** Ido Azuri, Ameer Wattad, Keren Peri-Hanania, Tamar Kashti, Ronnie Rosen, Yaron Caspi, Majdolen Istaiti, Makram Wattad, Yaakov Applbaum, Ari Zimran, Shoshana Revel-Vilk, Yonina C. Eldar

**Affiliations:** 1 Bioinformatics Unit, Department of Life Sciences Core Facilities, Weizmann Institute of Science, Rehovot 7610001, Israel; 2 Department of Radiology, Shaare Zedek Medical Center, Jerusalem 9103102, Israel; 3 Department of Computer Science and Applied Mathematics, Weizmann Institute of Science, Rehovot 7610001, Israel; 4 Gaucher Unit, Shaare Zedek Medical Center, Jerusalem 9103102, Israel; 5 Faculty of Medicine, Hebrew University, Jerusalem 9112102, Israel

**Keywords:** Gaucher disease, spleen volume, deep learning

## Abstract

The enlargement of the liver and spleen (hepatosplenomegaly) is a common manifestation of Gaucher disease (GD). An accurate estimation of the liver and spleen volumes in patients with GD, using imaging tools such as magnetic resonance imaging (MRI), is crucial for the baseline assessment and monitoring of the response to treatment. A commonly used method in clinical practice to estimate the spleen volume is the employment of a formula that uses the measurements of the craniocaudal length, diameter, and thickness of the spleen in MRI. However, the inaccuracy of this formula is significant, which, in turn, emphasizes the need for a more precise and reliable alternative. To this end, we employed deep-learning techniques, to achieve a more accurate spleen segmentation and, subsequently, calculate the resulting spleen volume with higher accuracy on a testing set cohort of 20 patients with GD. Our results indicate that the mean error obtained using the deep-learning approach to spleen volume estimation is 3.6 ± 2.7%, which is significantly lower than the common formula approach, which resulted in a mean error of 13.9 ± 9.6%. These findings suggest that the integration of deep-learning methods into the clinical routine practice for spleen volume calculation could lead to improved diagnostic and monitoring outcomes.

## 1. Introduction

Gaucher disease (GD) is a hereditary lysosomal storage disorder that is characterized by the accumulation of glucocerebroside in various organs, such as the spleen, liver, kidneys, lungs, brain, and bone marrow [1,2,3,4]. Common manifestations of GD include hepatosplenomegaly, anemia, thrombocytopenia, and skeletal abnormalities. The current therapies for GD, i.e., enzyme replacement therapy and substrate reduction therapy, can decrease the liver and spleen enlargement, and reverse liver and spleen fatty textures, to some extent. The therapy has shown encouraging outcomes in enhancing the quality of life of affected individuals [5,6,7,8,9].

A precise estimation of the liver and spleen volumes is a crucial aspect of the baseline assessment and monitoring of the treatment response in patients with GD. In this regard, magnetic resonance imaging (MRI) is a widely utilized imaging modality [10,11]. Radiologists commonly rely on a method that estimates the spleen volume from MRI images by measuring three distances; namely, the cranio-caudal distance between the first and last MRI slices in the axial plane where the spleen is depicted (L), the largest measurable long-axis diameter in the axial plane (D), and the largest perpendicular dimension to D in the axial plane (T). The formula employed to calculate the spleen volume is (cm^3^) = 30 + 0.58 × L × D × T, or equivalent formulae [8,9,10]. In some studies, this method has demonstrated a relatively high level of accuracy, with a mean error of 6% in the spleen volume calculation in the testing patient cohorts, and a strong correlation of the axial measurements with the spleen volume [12,13]. However, in other studies, especially in cases involving individuals with splenomegaly or spleen disorders, this method has been shown to produce relatively large errors, with a low correlation between the axial measurements and the spleen volume [14,15,16], as the shape of the spleen can significantly deviate from the formula of volume–shape approximation. Alternatively, for an accurate spleen volume estimation, manual segmentation should be performed by a radiologist for the spleen for each MRI slice, which has been demonstrated to be time-consuming and labor-intensive [16,17,18]. Consequently, there is a pressing need for a more accurate, consistent, and time-efficient approach to estimating liver and spleen volumes in patients with GD.

In recent years, the use of deep-learning methods in numerous and various fields has expanded, due to the increase in data availability, computing capability, and algorithmic improvements [19]. “Deep learning” refers to a set of algorithms in machine learning, based on artificial neural networks (ANNs). ANNs have been shown to exhibit superior performance in various tasks, including image segmentation, which refers to the process of classifying image pixel values based on their corresponding object class.

Deep learning in biomedical applications has been utilized successfully, in various studies, for abdominal organ segmentation [15,16,17,18,20,21,22,23,24,25,26,27,28,29,30,31,32,33,34,35,36,37,38,39]. In the present study, our focus is to employ deep-learning techniques for the precise segmentation of the spleen, and the calculation of the spleen volume, using the MRI scans of 20 patients with GD as a testing cohort.

We estimated the accuracy of the proposed method in comparison with the commonly used spleen volume formula method. To the best of our knowledge, this is the first time such a comparison has been carried out for MRI scans. Additionally, while there are only a few studies on spleen segmentation in splenomegaly cases, we further established the necessity of applying deep-learning methods for spleen volume calculation in a patients’ group with spleen disorders. To the best of our knowledge, this is the first work applying deep-learning methods for patients with GD. We show that the deep-learning method outperforms the commonly used spleen volume formula, and leads to a much higher accuracy.

## 2. Methods

### 2.1. MRI Imaging

Abdominal MRI scans of 1.5 Tesla (Siemens, Munich, Germany) of 30 patients with GD were included in the study. All patients were followed in the Gaucher Unit, Shaare Zedek Medical Center (SZMC), and had an abdominal MRI assessment of spleen volume performed at the Department of Radiology, SZMC, as part of clinical routine practice. The cases included in the study were randomly selected from a cohort of 100 cases. Abdominal MRI scans that had low-quality images due to technical issues, or that included additional abdominal pathologies not related to GD, were excluded from the study.

Picture Archiving and Communication System (PACS) was utilized to extract the axial out-of-phase T1 sequence with DICOM and TIFF image files. The MRI slice thickness was 3 mm. The boundaries of the spleen were marked on the TIFF file images by a radiology resident (A.W.), with the help of a computer science student (M.W.). All MRI scans were anonymized and coded prior to transferring them to the SAMPL Lab. The study was approved by the SZMC IRB; protocol number: 0520-20-SZMC. A waiver was received for the signing of informed consent.

### 2.2. Reference Labeling and Dataset

Among the 30 patients who underwent MRI, it was determined that a total of 20 patients’ MRI scans contained all the required slices, rendering them suitable for use as the test set. Two MRI scans had missing slices from the beginning or end of the spleen. Hence, volume evaluation could not be performed, and these MRIs were disqualified from inclusion in the testing set. In three MRIs, some of the outlined spleen slices were not accurate enough, or there were technical coding difficulties in extracting the masks from them. This created “sequence holes” in the scan, which could lead to an inaccurate estimation of the spleen volume. As a result, these MRIs were also disqualified from the testing set. Five additional MRI scans were provided at different resolutions and in different file settings to the other MRIs. As different resolutions and file settings may affect differently the accuracy metrics, it was decided to exclude these scans, so that the reported accuracy metric would not be affected positively by this bias.

**Figure 1:**
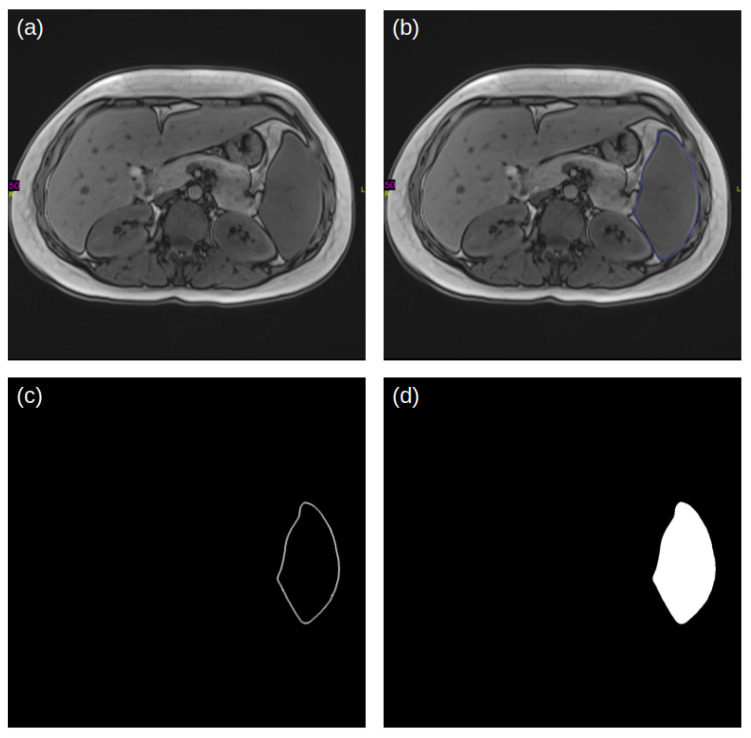
Example of an MRI slice (**a**), and the manual outline of the spleen in the purple color on the MRI slice (**b**). The pure outline of the spleen is extracted and shown in (**c**), and the filled outline (**d**) creates the mask for the spleen.

A manual segmentation process was employed to outline the boundaries of the spleen in the MRI slices (see MRI imaging in the Section 2). For each slice, a pair of images was generated; one excluded the outlined boundaries, while the other included the boundaries (Figure 1a,b, respectively). The boundaries were extracted from the pair of images (Figure 1c), and filled with the label value (in this case, 1, the white color when the background is 0, the black color), to yield the mask that corresponded to the spleen in the given slice (Figure 1d). These extracted masks served as the ground truth (GT) label for the spleen in each slice. The original MRI slices and the corresponding spleen labels were compiled, to create a dataset that was preprocessed prior to deep-learning modeling. The dataset consisted of 30 MRI scans and ~30–90 MRI slices containing the spleen per patient, resulting in a total of 1622 slices. The full scan for each patient contained ~100 slices.

### 2.3. Pre-Processing

Pre-processing is a crucial stage in classical image processing that precedes the deep-learning modeling. Its purpose is to enhance the model’s robustness to diverse inputs, and improve the accuracy performance. In the present study, MRI scans underwent three pre-processing operations. Firstly, the MRI scans were standardized (via patient scan mean subtraction and standard deviation division) and normalized (the standardized patient scan values were transferred to be between 0 and 1), to ensure that the image tissue features were comparable across the different MRI patient scans, and scaled consistently. Importantly, the patient scan mean and standard deviation were calculated for the body scan regions with the background excluded, in order to reduce the body size effect, and eliminate the background effect. Secondly, the slices were cropped (from the bottom-right corner of the slice, to ensure that the spleen was captured) to have a size of 256 × 256 pixels. This helped to reduce redundant data, and enhance the modeling efficiency. Finally, the slices were augmented (by rotations of ±5 degrees, only in the training set, tripling its number of slices) to generate more synthetic data, which has been demonstrated to improve accuracy, and mitigate overfitting [40].

### 2.4. Deep-Learning Modeling for Automated Segmentation

The pre-processed dataset was utilized to train a deep-learning model, with the spleen masks as the target. A total of 30 patient scans were used, with one patient scan excluded at a time for the testing set, while the remaining patient scans were used for training, utilizing a leave-one-out cross-validation scheme (see also the Section 2.5 below). The learning rate was optimized against the training dataset using 5-fold cross-validation. It is important to note that image augmentation was only applied to the training set.

A 2D U-Net model was employed for the segmentation, as U-Net-based models have been shown to outperform other models in various biomedical applications [41]. Our 2D U-Net consists of an encoder (image feature extraction) that down-samples the input image three times via a pooling operation, and a decoder (connecting the extracted image features) that up-samples the extracted image features, and yields the original size of the image. The model also has skip connections between matched encoder–decoder layers that make the modeling more robust, and lead to a higher accuracy. We used the Segmentation Models package 0.3.1 [42] in Pytorch 1.10.0 [43], with U-Net architecture and the resnet34 encoder. The encoder consists of four layers, and each layer is composed of a set of convolutions, batch normalization, and activation operations.

### 2.5. Testing Dataset

In order to evaluate the performance of the model on the testing set, we conducted a leave-one-out cross validation scheme on all 30 patients. Through this scheme, we trained the model 30 times. In the first iteration, the first patient was left out of the testing, and the remaining 29 patients were used for training. In the next iteration, the second patient was left out of the testing, and the remaining 29 patients were used for training, and so on, until all 30 patients had been used for testing. Practically, only 20 of the patients had full spleen slice GTs that were accurately detected and segmented during the preparation of the GT. Therefore, we applied the leave-one-out cross validation scheme on all 30 patients, but the reported results are solely for the 20 patients that constituted our testing dataset.

### 2.6. Post-Processing

**Figure 2:**
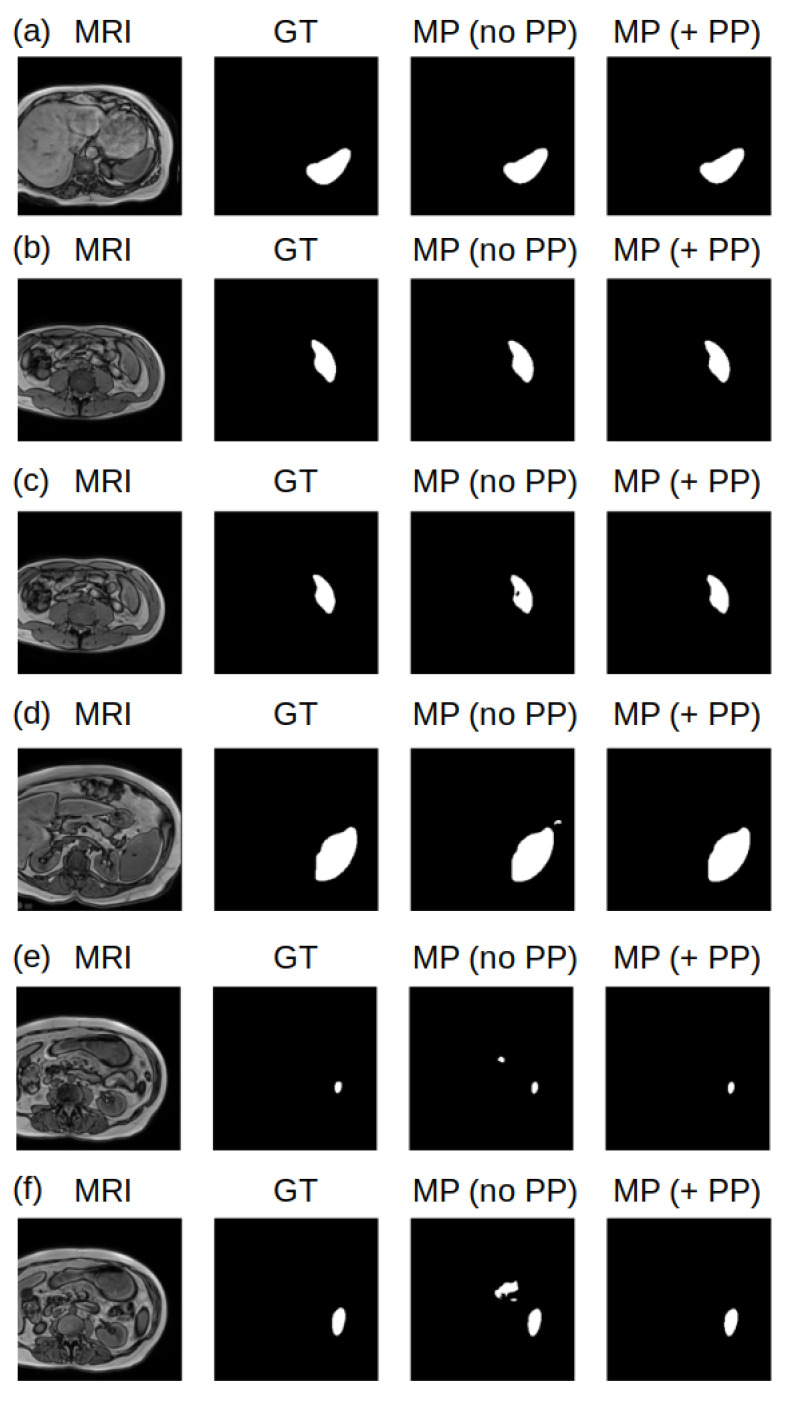
Examples of MRI slices (first column), their GT (second column), the MP (no PP) (third column), and the MP (+PP) (fourth column) for valid MP cases (**a**,**b**), filling holes (**c**), and removing inaccurate predictions (**d**–**f**).

Post-processing (PP) pipelines are an essential part of the deep-learning pipeline, to correct inaccurate model predictions (MP). In this study, we integrated several PP algorithmic steps into our pipeline. The first step involved filling holes in the MP segments that contained holes. The second step was connected component analysis. This operation enables the detection and labeling of all segments predicted by the model for a given slice as different components. The third step was the component size evaluation from the previous step. This operation enabled us to keep the most probable segment, which was the largest component (in pixel area) in the given slice (which should be associated with the spleen), and to remove noisy and inaccurate predictions (usually small-pixel-area components). The PP pipeline ensured the correct segmentation of the spleen, leading to a more precise volume calculation. Importantly, in this study, most of the MP slices were valid, and the PP did not have any effect on it, as in Figure 2a,b. Figure 2c shows an example in which the MP had a hole, and the hole-filling PP step corrected it. Figure 2d–f shows a case of MP artifacts, and the PP steps of connected component analysis and component size evaluation remove the MP artifacts. In this manuscript, we refer to MP as the MP (+PP) pipeline.

### 2.7. Spleen Volume Calculation

The spleen volume calculation is performed as follows. For each slice, the area of the spleen is calculated by summing the pixels associated with the spleen, and multiplying the result by the pixel size conversion factor. This gives the area of the spleen in the given slice. Then, the areas of all the slices are summed to yield the total volume of the spleen. For accurate summation, a trapezoid integration method is applied to integrate the areas with respect to the depth–size factor, which can be obtained from the DICOM file of each slice.

### 2.8. Full-Scan Spleen Volume Calculation

At the beginning and end of an MRI scan, the spleen is not presented. When a deep-learning model is applied to a slice where the spleen is not presented, ideally, it returns zero values for all pixels, signifying the absence of spleen detection. However, it is noteworthy that the model can predict artifacts in empty slices. That may decrease the accuracy of the calculated spleen volume, especially when the number of slices in which spleen in not presented can be large (more than 50% of the MRI scan). In order to improve the model performance, the region to which the spleen is confined (the perpendicular axis to the plane of the slices in the scanning direction), should be detected. Subsequently, the slices in which the model predicts artifacts outside of the confined region of the spleen should be nullified. One approach to this is to calculate the area of the predicted segment in each slice (slice plane). This returns a profile of the slices area as a function of the scanning direction. The ideal shape of such a profile starts with the line at the zero value (indicating spleen absence), then, when the spleen is detected, the value increases, up to a maximum value (when the spleen within a given slice is largest), and then decreases again, until there is no spleen, and ends with a line of zero values (spleen absence). Practically, in the zero-value lines, outside of the confined region of the spleen, some noise appears, due to the model artifact predictions. Moving from the maximum value on the profile to the right/left of the scanning direction will result in the first zero prediction. Then, from that point to the right/left, all the slices for which the model predicts artifacts should be nullified. This approach may be problematic if the first zero occurs far from the true confined region of the spleen. Alternatively, the edges of the region where the spleen is confined (the spleen is small there) can be detected via low-value thresholding on the area profile. This yields a set of slice indices corresponding to the locations of the right and left boundaries of the spleen in the scanning direction. Clustering these indices into two clusters yields two indices, corresponding to the right and left boundaries of the spleen, and representing the confined region of the spleen. Then, all the slices outside of the confined region should be nullified. We applied this approach in this study.

### 2.9. Dice Coefficient

In addition to the spleen volume calculation, we also calculated an accuracy metric known as the DC. The DC provides an indication of the overlap between the GT spleen segments and the MP segments, and is given by:

(1) $$\frac{2|GT\cap MP|}{\left(\left|GT\right|+|MP|\right)}.$$

When the Dice coefficient equals 1, it represents the best matching between the GT and MP, which means that the model has succeeded in accurately segmenting the spleen, and when the Dice coefficient equals 0, it means there is no matching at all between the GT and MP, and indicates a low model performance.

### 2.10. Software

All the code used in this study was implemented using Python 3 [44]. In addition to the default Python packages, several external libraries and packages were used, including PyTorch 1.10.0 [43] and Pytorch Segmentation Models 0.3.1 [42] for the deep-learning modeling, OpenCV 4.6.0 [45], SciPy.ndimage 1.9.1 [46], and scikit-image 0.19.2 [47] for image processing and manipulation, PIL 9.2.0 [48] and celluloid 0.2.0 [49] for the visualization of video, and Pydicom 2.3.1 for reading the DICOM files and obtaining the necessary patient field data [50].

## 3. Results

### 3.1. Modeling Pipeline

**Figure 3:**
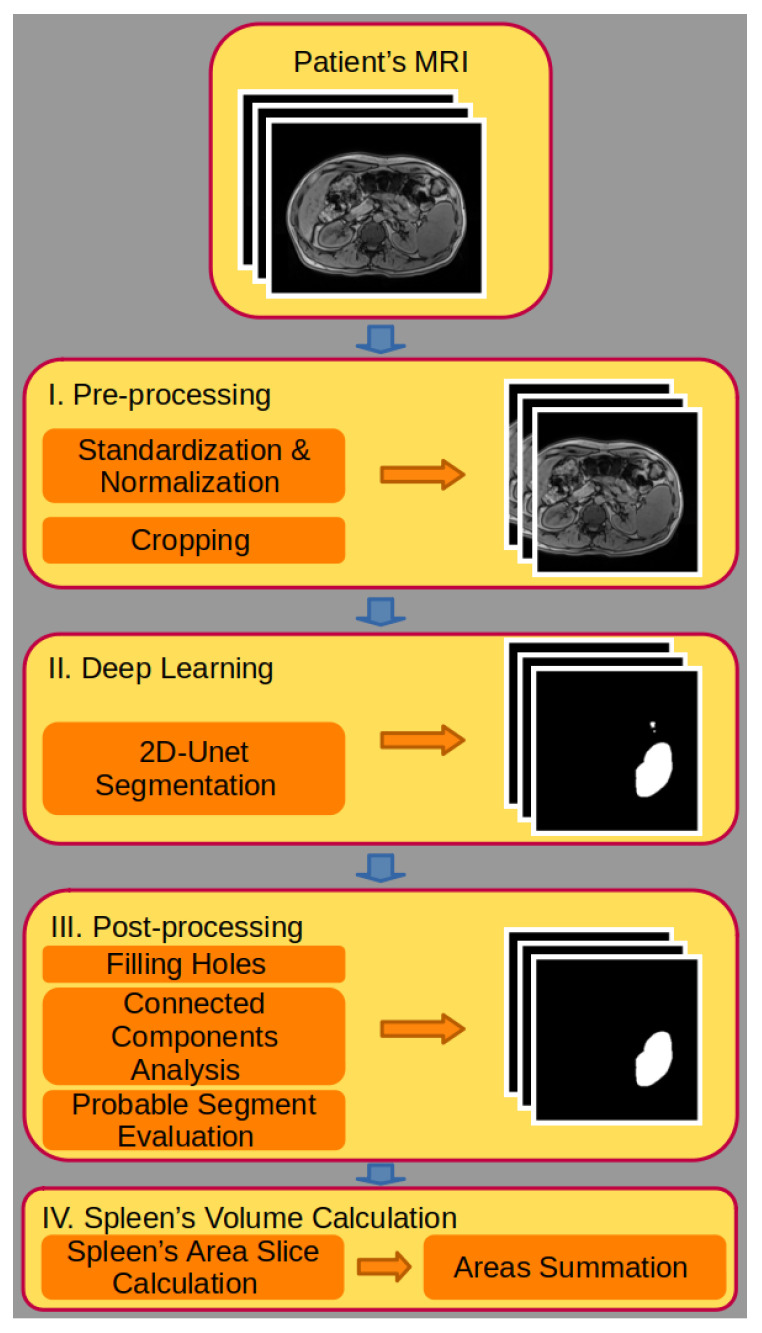
Schematic illustration of the algorithmic pipeline applied to the spleen volume calculation for the patient MRI scan, as explained in Section 2 and Section 3.1. The pipeline is composed of four steps: I. pre-processing the input MRI scan, II. the deep-learning segmentation of the spleen, III. the post-processing pipeline to correct MP slices that contain artifacts, and IV. the spleen volume calculation from the spleen MP slices.

Our modeling pipeline consists of pre-processing the MRI scans [51], inputting them into a deep-learning model that outputs the segmented spleen in each slice, and post-processing (PP) steps to correct inaccurate predictions [52] (Figure 3). The various steps are described in detail in the Section 2. Finally, the area of the spleen is calculated for each slice via pixel summation, followed by the integration of the areas, to yield the spleen volume (Figure 3).

### 3.2. Model Accuracy

In order to quantify the accuracy of the model, ground truth (GT) segmentation of the spleen was performed by a radiologist, and was compared to that obtained via the deep-learning model. We used the Dice coefficient (DC, see the relevant part in Methods for the definition of the DC) to indicate the amount of overlap between the GT and model segmentation. For a perfect segmentation, when the GT exactly matches the model prediction, a value of 100% is obtained. In the opposite case, for a completely inaccurate segmentation, where there is no overlap at all, a value of 0% is obtained. Values that are closer to 100% indicate a high accuracy performance of the model. The DC for the 20-patient testing set cohort was almost perfect (Table 1).

### 3.3. Spleen Volume Calculation

**Figure 4:**
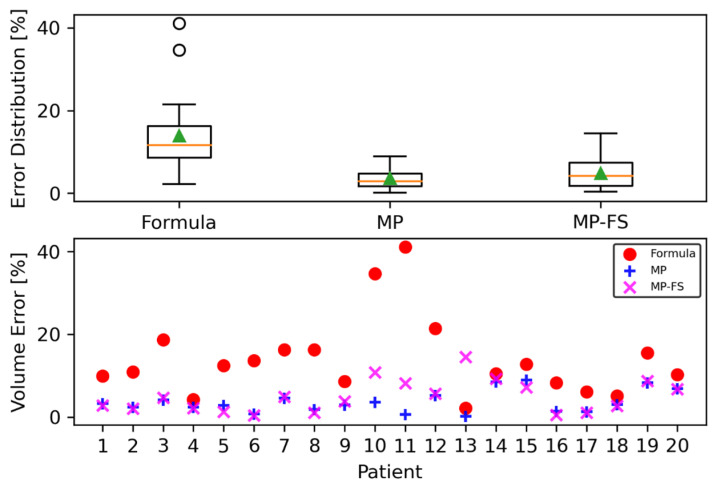
Spleen volume errors, with respect to the ground truth volumes. Upper panel: box plots for the formula, MP, and MP-FS methods. The box boundaries are the first quartile (Q1) and the third quartile (Q3) of the data, with the orange line representing the median. The green triangle is the average, and the whiskers extend from the box by 1.5×, the inter-quartile range (IQR: Q3–Q1). The outlier points are those past the end of the whiskers. Bottom panel: the spleen volume error for each patient, obtained using the formula, MP, and MP-FS methods. Clearly, the MP techniques outperform the traditional formula.

Table 2 and Figure 4 present the results of the spleen volume error with respect to the GT volumes for the two methods: the commonly used method for spleen volume estimation by radiologists (spleen volume formula), and the model prediction (MP) method. We also calculated the spleen volume, using the MP method, for the full MRI scan (MP-FS), where the spleen is not presented at the beginning and the end of the scan.

The results show that the MP yielded a mean relative error that was much smaller than the mean relative error obtained via the formula method. The standard deviation error was also smaller using the MP method. For two patients (numbers 10 and 11, Figure 4), where the GT volume was the smallest, and less than 350 cm^3^ (while all the other volumes were in the range of 450 cm^3^–2470 cm^3^), the error in the volume calculation using the formula method was relatively larger, more than 34%, while the error was much smaller—at most, 3.6%—using the MP methods. This suggests that the MP method is also valid for the smaller spleen volumes. Even after excluding these patients, the mean relative error was still large for the formula method, at 11.2%, compared to the MP method, demonstrating the advantages and consistency of the deep-learning-method predictions.

## 4. Discussion

According to the results presented in the review by Lenchik et al. [39], most of the studies of abdominal organ segmentation in radiology images were performed using CT (57%), and fewer using MRI scans (41%). The most segmented organs were the prostate, liver, and kidneys. The spleen ranked only in fourth place. In [39], most automated techniques were atlas-based (first place), followed by deformable and deep-learning models (second place). In a recent review [18] that compares the most segmented organs (the liver, kidneys, and spleen) using deep-learning methods, the spleen is the least segmented organ. In addition, there are far fewer studies about deep-learning segmentation in splenomegaly [15,25,26,27,28,29,32], and only a few of them using MRI [26,27].

A variety of deep-learning algorithms have been applied for spleen segmentation, including 2D and 3D U-Net based models, some of which combine a post-processing pipeline [15,16,17,20,22,23,25,26,28,29,32,33,34,35,36,37,38]. More advanced methods include the use of transformers [24,31]. In addition, researchers even designed a deep-learning neural network especially for spleen segmentation [27,30]. Most of the studies were carried out using CT [15,16,17,20,22,23,24,25,28,29,31,32,34,35,37], and the accuracy (DC) obtained in these studies was mostly > 95.0%. When compared to MRI studies [24,26,27,33,34,36,38], it was found that MRI in general is less accurate, and the top-range accuracy is mostly around 94%.

Our study is the first one that uses deep learning to calculate the spleen volume using MRI of patients with GD. As the spleen size varies between patients with GD, and changes with treatment, a reliable prediction model that is accurate both for a smaller and larger spleen is important. In addition, this is the first time that deep-learning spleen volumes are compared to those obtained using the formula in an MRI study. The deep-learning methods resulted in a significantly lower relative error and standard deviation in the prediction of spleen volume, compared to the commonly used formula.

An accurate spleen volume calculation may also serve as an accurate diagnostic tool. This is very important in the early stages of the disease. According to Simon et al. [53], the first symptoms of the disease occur before 10 years of age in at least 50% of the patients. An early diagnosis of the disease is effective in stopping the disease progression, leads to the regression of abnormalities, prevents irreversible bone deformities, and improves quality of life.

We aim to deploy the deep-learning pipeline for in-house use, for fast and accurate spleen volume estimation. Through this, and the high accuracy obtained, we will also be able to track spleen volume changes in patients with GD. We do not expect the model to be generalized to MRI scans that come from different scanners or apparatuses [36,37]. In addition, it was shown in [37] that deep-learning models that are customized for in-house applications yield a much better performance than models that are trained on many different datasets, and used on other datasets for segmentation.

Finally, the model prediction method for the full MRI scan (MP-FS), where the spleen is not presented at the beginning and end of the scan, may serve as a true indication of the MP’s performance. Only in one patient (number 13, Figure 4) the error is larger than that of the formula method. Overall, the average error is low, at 4.9%, with a standard deviation of 3.9%, and it still outperformed the formula method.

## 5. Conclusions

The proposed method has the potential to be applied and utilized by physicians for the accurate diagnosis of spleen disorders, and to monitor the response to treatment. Furthermore, the results of this study demonstrate the potential of deep-learning methods in accurate and efficient spleen segmentation and volume calculation in Gaucher patients. While the development of the deep-learning model and algorithmic solution may require significant time and labor, the automatic and efficient nature of the method makes it a valuable tool for clinical routine and in-house applications. However, it is important to note that these methods need to be updated and re-trained, to account for changes in MRI data, and to work with different MRI machines.

## Figures and Tables

**Table 1:** Dice coefficient (DC) for the 20-patient testing set cohort.

	DC [%]
Mean	94.1
Standard deviation	1.9
Range	89.6–96.8

**Table 2:** Relative error in the spleen volume calculation, with respect to the ground truth volume, for the 20-patient testing set cohort.

	Formula [%]	Model Prediction [%]	Model Prediction—Full Scan [%]
Mean	13.9	3.6	4.9
Standard deviation	9.6	2.7	3.9
Range	2.2–41.2	0.12–8.91	0.39–14.5

## Data Availability

The data are not publicly available due to the privacy of the patients.

## References

[1] Revel-Vilk S., Szer J., Zimran A., Kaushansky K., Lichtman M., Prchal J., Levi M., Burns L. (2021). Gaucher disease and related lysosomal storage diseases. Williams Hematology.

[2] Bennett L.L., Mohan D. (2013). Gaucher disease and its treatment options. Ann. Pharmacother..

[3] Stirnemann J., Belmatoug N., Camou F., Serratrice C., Froissart R., Caillaud C., Levade T., Astudillo L., Serratrice J., Brassier A. (2017). A Review of Gaucher Disease Pathophysiology, Clinical Presentation and Treatments. Int. J. Mol. Sci..

[4] Nalysnyk L., Rotella P., Simeone J.C., Hamed A., Weinreb N. (2017). Gaucher disease epidemiology and natural history: A comprehensive review of the literature. Hematology.

[5] Revel-Vilk S., Szer J., Mehta A., Zimran A. (2018). How we manage Gaucher Disease in the era of choices. Br. J. Haematol..

[6] Guggenbuhl P., Grosbois B., Chalès G. (2008). Gaucher disease. Jt. Bone Spine.

[7] Futerman A.H., Sussman J.L., Horowitz M., Silman I., Zimran A. (2004). New directions in the treatment of Gaucher disease. Trends Pharmacol. Sci..

[8] Nagral A. (2014). Gaucher disease. J. Clin. Exp. Hepatol..

[9] Revel-Vilk S., Szer J., Zimran A. (2021). Hematological manifestations and complications of Gaucher disease. Expert. Rev. Hematol..

[10] Robertson F., Leander P., Ekberg O. (2001). Radiology of the spleen. Eur. Radiol..

[11] Linguraru M.G., Sandberg J.K., Jones E.C., Summers R.M. (2013). Assessing splenomegaly: Automated volumetric analysis of the spleen. Acad. Radiol..

[12] Prassopoulos P., Daskalogiannaki M., Raissaki M., Hatjidakis A., Gourtsoyiannis N. (1997). Determination of normal splenic volume on computed tomography in relation to age, gender and body habitus. Eur. Radiol..

[13] Bezerra A.S., D’Ippolito G., Faintuch S., Szejnfeld J., Ahmed M. (2005). Determination of splenomegaly by CT: Is there a place for a single measurement?. AJR Am. J. Roentgenol..

[14] Nuffer Z., Marini T., Rupasov A., Kwak S., Bhatt S. (2017). The Best Single Measurement for Assessing Splenomegaly in Patients with Cirrhotic Liver Morphology. Acad. Radiol..

[15] Humpire-Mamani G.E., Bukala J., Scholten E.T., Prokop M., van Ginneken B., Jacobs C. (2020). Fully automatic volume measurement of the spleen at CT using deep learning. Radiol. Artif. Intell..

[16] Moon H., Huo Y., Abramson R.G., Peters R.A., Assad A., Moyo T.K., Savona M.R., Landman B.A. (2019). Acceleration of spleen segmentation with end-to-end deep learning method and automated pipeline. Comput. Biol. Med..

[17] Sharbatdaran A., Romano D., Teichman K., Dev H., Raza S.I., Goel A., Moghadam M.C., Blumenfeld J.D., Chevalier J.M., Shimonov D. (2022). Deep Learning Automation of Kidney, Liver, and Spleen Segmentation for Organ Volume Measurements in Autosomal Dominant Polycystic Kidney Disease. Tomography.

[18] Altini N., Prencipe B., Cascarano G.D., Brunetti A., Brunetti G., Triggiani V., Carnimeo L., Marino F., Guerriero A., Villani L. (2022). Liver, kidney and spleen segmentation from CT scans and MRI with deep learning: A survey. Neurocomputing.

[19] Bengio Y., Goodfellow I., Courville A. (2017). Deep Learning.

[20] Ma J., Zhang Y., Gu S., Zhu C., Ge C., Zhang Y., An X., Wang C., Wang Q., Liu X. (2021). AbdomenCT-1K: Is Abdominal Organ Segmentation A Solved Problem?. IEEE Trans. Pattern Anal. Mach. Intell..

[21] Zhou S.K., Greenspan H., Davatzikos C., Duncan J.S., van Ginneken B., Madabhushi A., Prince J.L., Rueckert D., Summers R.M. (2021). A review of deep learning in medical imaging: Image traits, technology trends, case studies with progress highlights, and future promises. Proc. IEEE.

[22] Conze P.H., Kavur A.E., Cornec-Le Gall E., Sinem Gezer N., Le Meur Y., Alper Selver M., Rousseau F. (2021). Abdominal multi-organ segmentation with cascaded convolutional and adversarial deep networks. Artif. Intell. Med..

[23] Tang Y., Gao R., Lee H.H., Han S., Chen Y., Gao D., Nath V., Bermudez C., Savona M.R., Abramson R.G. (2021). High-resolution 3D abdominal segmentation with random patch network fusion. Med. Image Anal..

[24] Hatamizadeh A., Tang Y., Nath V., Yang D., Myronenko A., Landman B., Roth H., Xu D. UNETR: Transformers for 3D Medical Image Segmentation. Proceedings of the 2022 IEEE/CVF Winter Conference on Applications of Computer Vision (WACV).

[25] Yang Y., Tang Y., Gao R., Bao S., Huo Y., McKenna M.T., Savona M.R., Abramson R.G., Landman B.A. (2021). Validation and estimation of spleen volume via computer-assisted segmentation on clinically acquired CT scans. J. Med. Imaging.

[26] Bobo M.F., Bao S., Huo Y., Yao Y., Virostko J., Plassard A.J., Lyu I., Assad A., Abramson R.G., Hilmes M.A. (2018). Fully Convolutional Neural Networks Improve Abdominal Organ Segmentation. Proc. SPIE Int. Soc. Opt. Eng..

[27] Huo Y., Xu Z., Bao S., Bermudez C., Plassard A.J., Liu J., Yao Y., Assad A., Abramson R.G., Landman B.A. (2018). Splenomegaly Segmentation using Global Convolutional Kernels and Conditional Generative Adversarial Networks. Proc. SPIE Int. Soc. Opt. Eng..

[28] Tang Y., Huo Y., Xiong Y., Moon H., Assad A., Moyo T.K., Savona M.R., Abramson R., Landman B.A. (2019). Improving Splenomegaly Segmentation by Learning from Heterogeneous Multi-Source Labels. Proc. SPIE Int. Soc. Opt. Eng..

[29] Ahn Y., Yoon J.S., Lee S.S., Suk H.I., Son J.H., Sung Y.S., Lee Y., Kang B.K., Kim H.S. (2020). Deep Learning Algorithm for Automated Segmentation and Volume Measurement of the Liver and Spleen Using Portal Venous Phase Computed Tomography Images. Korean J. Radiol..

[30] Huo Y., Xu Z., Bao S., Bermudez C., Moon H., Parvathaneni P., Moyo T.K., Savona M.R., Assad A., Abramson R.G. (2019). Splenomegaly Segmentation on Multi-Modal MRI Using Deep Convolutional Networks. IEEE Trans. Med. Imaging.

[31] Yan Q., Liu S., Xu S., Dong C., Li Z., Shi Javen Q., Zhang Y., Dai D. (2023). 3D Medical image segmentation using parallel transformers. Pattern Recognit..

[32] Meddeb A., Kossen T., Bressem K.K., Molinski N., Hamm B., Nagel S.N. (2022). Two-Stage Deep Learning Model for Automated Segmentation and Classification of Splenomegaly. Cancers.

[33] Chen Y., Ruan D., Xiao J., Wang L., Sun B., Saouaf R., Yang W., Li D., Fan Z. (2020). Fully automated multiorgan segmentation in abdominal magnetic resonance imaging with deep neural networks. Med. Phys..

[34] Valindria V.V., Pawlowski N., Rajchl M., Lavdas I., Aboagye E.O., Rockall A.G., Rueckert D., Glocker B. Multi-modal Learning from Unpaired Images: Application to Multi-organ Segmentation in CT and MRI. Proceedings of the IEEE Winter Conference on Applications of Computer Vision (WACV) 2018.

[35] Müller L., Kloeckner R., Mähringer-Kunz A., Stoehr F., Düber C., Arnhold G., Gairing S.J., Foerster F., Weinmann A., Galle P.R. (2022). Fully automated AI-based splenic segmentation for predicting survival and estimating the risk of hepatic decompensation in TACE patients with HCC. Eur. Radiol..

[36] Rickmann A.M., Senapati J., Kovalenko O., Peters A., Bamberg F., Wachinger C. (2022). AbdomenNet: Deep neural network for abdominal organ segmentation in epidemiologic imaging studies. BMC Med. Imaging.

[37] Meddeb A., Kossen T., Bressem K.K., Hamm B., Nagel S.N. (2021). Evaluation of a Deep Learning Algorithm for Automated Spleen Segmentation in Patients with Conditions Directly or Indirectly Affecting the Spleen. Tomography.

[38] Park H.J., Yoon J.S., Lee S.S., Suk H.I., Park B., Sung Y.S., Hong S.B., Ryu H. (2022). Deep Learning-Based Assessment of Functional Liver Capacity Using Gadoxetic Acid-Enhanced Hepatobiliary Phase MRI. Korean J. Radiol..

[39] Lenchik L., Heacock L., Weaver A.A., Boutin R.D., Cook T.S., Itri J., Filippi C.G., Gullapalli R.P., Lee J., Zagurovskaya M. (2019). Automated Segmentation of Tissues Using CT and MRI: A Systematic Review. Acad. Radiol..

[40] Krizhevsky A., Sutskever I., Hinton G.E. (2012). ImageNet Classification with Deep Convolutional Neural Networks. Adv. Neural Inf. Process. Syst..

[41] Ronneberger O., Fischer P., Brox T. (2015). U-Net: Convolutional Networks for Biomedical Image Segmentation. arXiv.

[42] Segmentation Models. https://segmentation-modelspytorch.readthedocs.io/en/latest/.

[43] Paszke A., Gross S., Massa F., Lerer A., Bradbury J., Chanan G., Killeen T., Lin Z., Gimelshein N., Antiga L. (2019). PyTorch: An Imperative Style, High-Performance Deep Learning Library. arXiv.

[44] Van Rossum G., Drake F.L. (1995). Python Reference Manual.

[45] Home–OpenCV. https://opencv.org.

[46] Virtanen P., Gommers R., Oliphant T.E., Haberland M., Reddy T., Cournapeau D., Burovski E., Peterson P., Weckesser W., Bright J. (2020). SciPy 1.0 Contributors. Nat. Methods.

[47] van der Walt S., Schönberger J.L., Nunez-Iglesias J., Boulogne F., Warner J.D., Yager N., Gouillart E., Yu T. (2014). and the scikit-image contributors. scikit-image: Image processing in Python. PeerJ.

[48] Umesh P. (2012). Image Processing in Python. CSI Commun..

[49] Celluloid. https://github.com/jwkvam/celluloid.

[50] Mason D. (2011). SU-E-T-33: Pydicom: An open source DICOM library. Med. Phys..

[51] Masoudi S., Harmon S.A., Mehralivand S., Walker S.M., Raviprakash H., Bagci U., Choyke P.L., Turkbey B. (2021). Quick guide on radiology image pre-processing for deep learning applications in prostate cancer research. J. Med. Imaging.

[52] Furtado P. (2021). Improving Deep Segmentation of Abdominal Organs MRI by Post-Processing. BioMedInformatics.

[53] Simon G., Erdos M., Maródi L., Tóth J. (2008). Gaucher-kór: A korai diagnózis és terápia jelentôsége [Gaucher disease: Importance of early diagnosis and therapy]. Orv. Hetil..

